# Behavior Change Techniques in Digital Health Interventions for Midlife Women: Systematic Review

**DOI:** 10.2196/37234

**Published:** 2022-11-09

**Authors:** Hana Sediva, Tina Cartwright, Claire Robertson, Sanjoy K Deb

**Affiliations:** 1 Centre for Nutraceuticals School of Life Sciences University of Westminster London United Kingdom; 2 School of Social Sciences University of Westminster London United Kingdom; 3 School of Life Sciences University of Westminster London United Kingdom

**Keywords:** menopause, midlife, women’s health, lifestyle, behavior change technique, BCT, behavioral intervention, digital health, mobile health, mHealth, menopausal symptom, behavior change, review, mobile phone

## Abstract

**Background:**

Digital health interventions are efficacious in health-promoting behaviors (eg, healthy eating and regular physical activity) that mitigate health risks and menopausal symptoms in midlife. However, integrated evidence-based knowledge about the mechanisms of change in these interventions is unclear.

**Objective:**

This systematic review aimed to evaluate studies on behavior change techniques (BCTs) and mechanisms of change in digital health interventions aimed at promoting health-enhancing behaviors in midlife women (aged 40-65 years).

**Methods:**

A systematic literature search of the electronic databases PubMed, Web of Science, PsycINFO, and Cochrane Central Register of Controlled Trials in the Cochrane Library was conducted. In total, 2 independent reviewers selected the studies for inclusion, extracted data, and completed BCT mapping of eligible studies. The mechanism of action and intervention functions of eligible studies were evaluated using the behavior change wheel framework. Reporting of psychological theory use within these interventions was explored using the Theory Coding Scheme. Mode of delivery, psychological theory, and BCTs were presented as descriptive statistics.

**Results:**

In total, 13 interventions (including 1315 women) reviewed used 13 (SD 4.30, range 6-21) BCTs per intervention on average. The “Shaping knowledge” and “Repetition and substitution” behavior change categories were used most frequently, with 92% (12/13) of the interventions implementing at least one of the BCTs from these 2 categories. Only 13.98% (169/1209) of the 93 available BCTs were used, with “Instructions on behaviour” most frequently used (12/13, 92%). The behavior change wheel mapping suggests that half of the intervention content aimed to increase “Capability” (49/98, 50% of the intervention strategies), “Motivation” (41/98, 42%), and “Opportunity” (8/98, 8%). “Behavioural Regulation” was the most frequently used mechanism of action (15/98, 15%), followed by increasing “Knowledge” (13/98, 13%) and “Cognitive and Interpersonal skills” (10/98, 10%). A total of 78% (7/9) of the intervention functions were used in the studies to change behavior, primarily through “Enablement” (60/169, 35.5%), whereas no study used “Restriction” or “Modelling” functions. Although 69% (9/13) of the interventions mentioned a psychological theory or model, most (10/13, 77%) stated or suggested rather than demonstrated the use of a theoretical base, and none reported explicit links between all BCTs within the intervention and the targeted theoretical constructs. Technological components were primarily based on web-based (9/13, 69%) modes of delivery, followed by phone or SMS text message (8/13, 62%) and wearables (7/13, 54%).

**Conclusions:**

The findings of this review indicate an overall weak use of theory, low levels of treatment fidelity, insignificant outcomes, and insufficient description of several interventions to support the assessment of how specific BCTs were activated. Thus, the identified limitations in the current literature provide an opportunity to improve the design of lifestyle health-enhancing interventions for women in midlife.

**Trial Registration:**

PROSPERO CRD42021259246; https://tinyurl.com/4ph74a9u

## Introduction

### Background

Approximately 3.5 million women aged 50 to 65 years are employed in the United Kingdom and experience menopausal symptoms (eg, hot flushes, disturbed sleep, depression, and cognitive dysfunction) [1] that can contribute to job dissatisfaction and decreased commitment to work [2]. The impact can be bidirectional, with symptoms such as poor concentration, poor memory, and sickness absence impairing job performance [3] and the workplace exacerbating menopausal symptoms [4]. Moreover, an individual’s health-related quality of life in midlife is influenced by many additional nonmenopausal factors such as lifestyle, physical activity (PA), and social integration [5]. Evidence suggests that midlife for women represents a critical window for preventing chronic disease and optimizing health and functioning, whereas it is increasingly recognized that a healthy lifestyle may mitigate such health risks [6]. Improvements in diet, PA, and lifestyle can provide an effective intervention to manage menopause symptoms, improve health-related quality of life [7], and reduce menopause-related health risks [8,9] (eg, neurodegenerative diseases, particularly Alzheimer disease [1,10], and increased cardiovascular disease risk [11], low bone-mineral density, fractures, and osteoporosis [12,13]).

Behavior change interventions (BCIs) aimed at promoting population-level participation in key behaviors have been widely applied in the general population [14] and, to some degree, also in midlife women [15,16]. Women in midlife are willing to make positive health behavior changes but need support (eg, social connectivity [17]) for those changes to be effective [18]. However, changing established behavior patterns can be challenging as it requires addressing a strong psychological, environmental, or social gradient [19]. BCIs are typically complex and involve many interacting components and, therefore, a theoretical understanding of how the intervention causes behavior change is needed to strengthen the effects of BCI on clinical outcomes [20]. A recent scoping review [21] identified limitations in describing PA interventions in midlife women, with only 59% of the 51 studies specifying an underlying theoretical model. Many studies provided a limited description of how behavior change techniques (BCTs) were activated to achieve desired outcomes and provided limited insight into how the BCTs were received by midlife women [21]. As a result, interpreting designs and evaluations of complex interventions can be challenging without sufficient description of key intervention content [22] and, therefore, characterizing interventions by BCTs can be insightful in understanding the effectiveness of interventions [23].

The use of psychological theory in the development of BCIs (including digital BCIs) is associated with greater intervention effects [24-26]. Although there is a wide range of theoretical models of behavior (eg, the theory of planned behavior [27] and the Health Belief Model [28]), health-promoting interventions that are based on a single theory have generally been shown to be more effective in changing behavioral intentions than actual behavior [29,30]. Therefore, integrated theories have been proposed to overcome this limitation by drawing their hypotheses from several different theories with the aim of providing a more comprehensive explanation of behavior [31]. Theoretical frameworks such as the Theoretical Domains Framework (TDF) [32] integrate insights of multiple behavioral theories to identify relevant constructs that may be implicated in various health behaviors. Together with the capability, opportunity, and motivation-behavior (COM-B) model [33] that aims to identify the sources of target behavior, they form the behavior change wheel (BCW) framework [19]. The BCW framework provides a systematic and theoretical basis for understanding and changing behavior [19]. It has been used extensively to develop and evaluate the implementation of interventions in health care settings [34-36] and lifestyle (eg, smoking cessation [37], alcohol use prevention [38], sedentary behavior [39], PA [40], and dietary patterns [41]) but also in other areas such as personal transportation habits [42].

Behavioral interventions to promote PA in midlife women have been traditionally delivered face-to-face or in group settings [43]. However, the use of digital technology to change health behaviors has increased exponentially in recent decades, primarily after the introduction of smartphones in 2009 [44]. Moreover, digital health technology (ie, apps, wearables, and websites) has the potential to increase scalability through broader user reach [43] throughout the day, improve intervention effectiveness [17], and achieve greater cost-efficiency [45,46]. Indeed, digital health interventions (DHIs) are both feasible [47,48] and acceptable among midlife women [17,49,50]. Digital health technologies (including therapeutic interventions, online support communities, and web-based consultations) can provide important means for midlife women to obtain evidence-based menopause-related health information and recommendations, social and health practitioner support, and symptom tracking [51].

### Objectives

The development of the BCT taxonomy [52,53] and methods for assessing the extent to which behavioral interventions are theory-based allows for more sophisticated coding of intervention content and insight into how and why the intervention promoted behavior change. Thus, the primary aim of this systematic review was to (1) assess the frequency and type of BCTs and BCT categories (representing groups of BCTs) used in DHIs with midlife women, (2) understand the mechanism of action proposed to affect changes in the behavioral outcome, and (3) appraise the intervention functions or broad categories of means by which the studies proposed to change behavior using the BCW. In addition, this review identified the theoretical grounding (or the extent of behavior change theory used) in the DHIs using the Theory Coding Scheme (TCS) [54] and determined the technological features (mode of delivery) used in these studies.

## Methods

The structure of this paper follows the PRISMA (Preferred Reporting Items for Systematic Reviews and Meta-Analyses) guidelines [55] as the basis for reporting findings from the selected trials. The study protocol was registered in PROSPERO (CRD42021259246).

### Selection Criteria

In accordance with PRISMA guidelines, the Population, Intervention, Comparison, Outcome, and Study Design tool was deployed.

#### Population

Women aged 40 to 65 years of all ethnicities and health conditions— including healthy women, those with overweight, and those with obesity—as well as survivors of breast cancer and women with a high risk of hypertension were included. These broad criteria were used to explore the impact of behavior change theory on lifestyle improvements rather than the interaction with these disease states.

#### Interventions

Studies describing interventions where the stated aim was to improve diet, PA, sleep, menopausal symptoms, and body composition by promoting changes in health behaviors, including healthy eating (single nutrients or whole dietary patterns) and PA (frequency or intensity), were considered. Only studies with participants randomized to a group that was explicitly asked to use digital technology (eg, wearables, mobile apps, and websites) as a mode of intervention delivery were considered. No other restrictions were placed on intervention type and delivery or duration.

#### Comparison

Control or other treatment groups involving health education, assignment of no digital health technology, or altered (ie, frequency or intensity) or no PA or diet intervention were included.

#### Outcome

The primary health outcomes were changes in PA (ie, frequency and intensity), diet (ie, fruit and vegetable intake and single nutrient intake), body composition (ie, body weight, lean muscle mass, and waist circumference), and frequency or intensity of menopausal symptoms (ie, vasomotor symptoms, sleep, bone health, anxiety, and depression). Although these health outcomes were included in the search criteria, they were not part of this review’s assessment of study designs (described in the study aims). However, when available, the outcomes of the interventions were extracted as part of the description of the studies to allow the main outcomes to be presented in the relevant context.

#### Study Design

Both experimental (ie, randomized controlled trials and quasi-experimental studies) and nonexperimental (ie, observational studies) studies were included, with a minimum of 2 arms for randomized controlled trials, pilot studies, and feasibility studies.

### Search Strategy

Literature searches were conducted by HS and SD between February 2021 and April 2021. Articles published before April 2021 and available in English were searched in the following databases: PubMed, Web of Science, PsycINFO, and Cochrane Central Register of Controlled Trials in the Cochrane Library. The search criteria included the following terms: (“midlife”) AND (“mHealth” OR “eHealth” OR “digital”) AND (“diet” OR “physical activity” OR “menopaus* symptom*” OR “lifestyle” OR “weight loss” OR “mental health” OR “depression” OR “sleep”). The filters used were “Randomised Controlled Trials” and “Clinical Trials;” the species selected was “Humans” only; and the language selected was “English” only. The search was limited to studies published between January 2009 and April 2021, reflecting the increased use of digital technology to change health behaviors after the introduction of smartphones in 2009 [44]. Interventions published before 2009 focusing on older technologies such as pedometers were not considered. Additional hand searches of relevant journals were performed, which included *JMIR mHealth and uHealth*, *Menopause*, and *Climacteric*.

### Data Extraction and Collection Process

The studies were screened using titles and abstracts, and those that did not meet the inclusion criteria were excluded. The following information was extracted from each study: author, behavior change theory, intervention type, study design, country, participant ethnicity, intervention length, participant age and health risk, comparison group, and significant between-group differences in main outcomes. Data from each eligible study were populated into a prepared Microsoft Excel template to evaluate their eligibility and observe any missing data. Two reviewers (HS and SD) independently extracted the data, and this was checked for accuracy. Any disagreements were resolved through discussion, and a third reviewer was not required.

The first template included key information on each study, such as the intervention type and length, behavior change theories used, outcomes, and mean age of the participants. Multiple studies from the same trial were merged, and information was extracted to gain a full picture of the intervention, ensuring that the reported descriptive statistics were not double-counted. The authors of the original reports were contacted to obtain further details if insufficient information was included in the published documents. A reminder was sent if no responses were received after 2 weeks.

The use of BCTs and clusters of BCTs as defined by the Behavior Change Technique Taxonomy v1 (BCTTv1 [53]) was synthesized (and coded) for each included study. The number of individual BCTs included in each study was counted (range 0-93), and the mean value and SD were reported. Furthermore, the use of behavior change categories and combinations of techniques and categories or clusters was investigated for each included study. Each study and group of related studies (ie, weight loss, lifestyle, and menopause symptoms) was mapped into this framework. The overarching synthesis bringing the studies together by providing a systematic and theoretical basis for understanding behavior was based on the COM-B model [33], TDF [32], and BCW [19]. The BCW links to theory-based frameworks (ie, the TDF and BCTTv1) to understand behavior for specifying intervention content [56]. Using the TDF or the COM-B model, intervention designers can make a behavioral diagnosis of what needs to change for the desired behavior to occur and, in the evaluation of interventions, the framework can help identify the mechanism of action (ie, how an intervention is working) [56]. Explicit links between the COM-B model and TDF domains are provided in the BCW guide [19].

The BCW also supports evaluating intervention functions (consisting of education, persuasion, incentivization, coercion, training, restriction, environmental restructuring, modeling, and enablement) by identifying broad categories of means by which interventions can change behavior [56]. For example, a digital health app designed to promote healthy eating may contain an educational element (eg, providing new information about the benefits of healthy eating) but may also be presented in a way that is intended to be persuasive (eg, generating feelings of worry about the health consequences of eating unhealthy foods) [56].

The use of psychological theory in the studies was examined using the TCS [54] to assess the extent to which behavior change theory was used to design the interventions in each study. These data were extracted by HS and reviewed for accuracy by SD. The overall score for each study was assessed as having weak (score 0-7), moderate (score 8-15), or strong (score 16-23) levels of theory use [57]. Finally, the technological and nontechnological components of each study were extracted and mapped into predefined categories that were created based on the review of the included studies. Frequencies of individual modes of delivery were reported together with the frequencies of related groups of passive and action-based components. The quality assessment was completed using the Physiotherapy Evidence Database scale [58], and the Cochrane risk-of-bias tool for randomized trials [59] was used to assess the risk of bias in randomized trials (these data are presented in Multimedia Appendix 1 [48,60-73]).

### Treatment Fidelity Assessment

Treatment fidelity facilitates theory testing, with high levels often associated with alterations in the mechanisms of change (eg, increased PA and healthier eating) hypothesized to affect the outcomes [74]. According to Borrelli [74], high fidelity constitutes 80% to 100% integrity, whereas 50% constitutes low-fidelity scoring. By describing methodological strategies that are applied to monitor and enhance the reliability and validity of health BCIs [75], treatment fidelity helps increase scientific confidence that the changes in the outcome of interest (dependent variable) are due to the manipulation of other variables (independent variables) by the researchers [74]. This is achieved through assessment of the degrees to which the intervention is implemented as intended and the study arms differ along critical dimensions [74]. In interventions that produce nonsignificant effects, treatment fidelity helps uncover whether these effects are due to the omission or addition of active or inactive components or to an ineffective treatment [74]. The treatment fidelity of the studies included in this review was assessed using a 29-item checklist [74] grouped into 5 domains. These are (1) design of study (6 items), (2) monitoring and improving provider training (7 items), (3) monitoring and improving delivery of treatment (9 items), (4) monitoring and improving receipt of treatment (5 items), and (5) monitoring and improving enactment of treatment skills (2 items) [75], termed henceforth study design, training, delivery, receipt, and enactment [41].

## Results

### Study Selection

**Figure 1:**
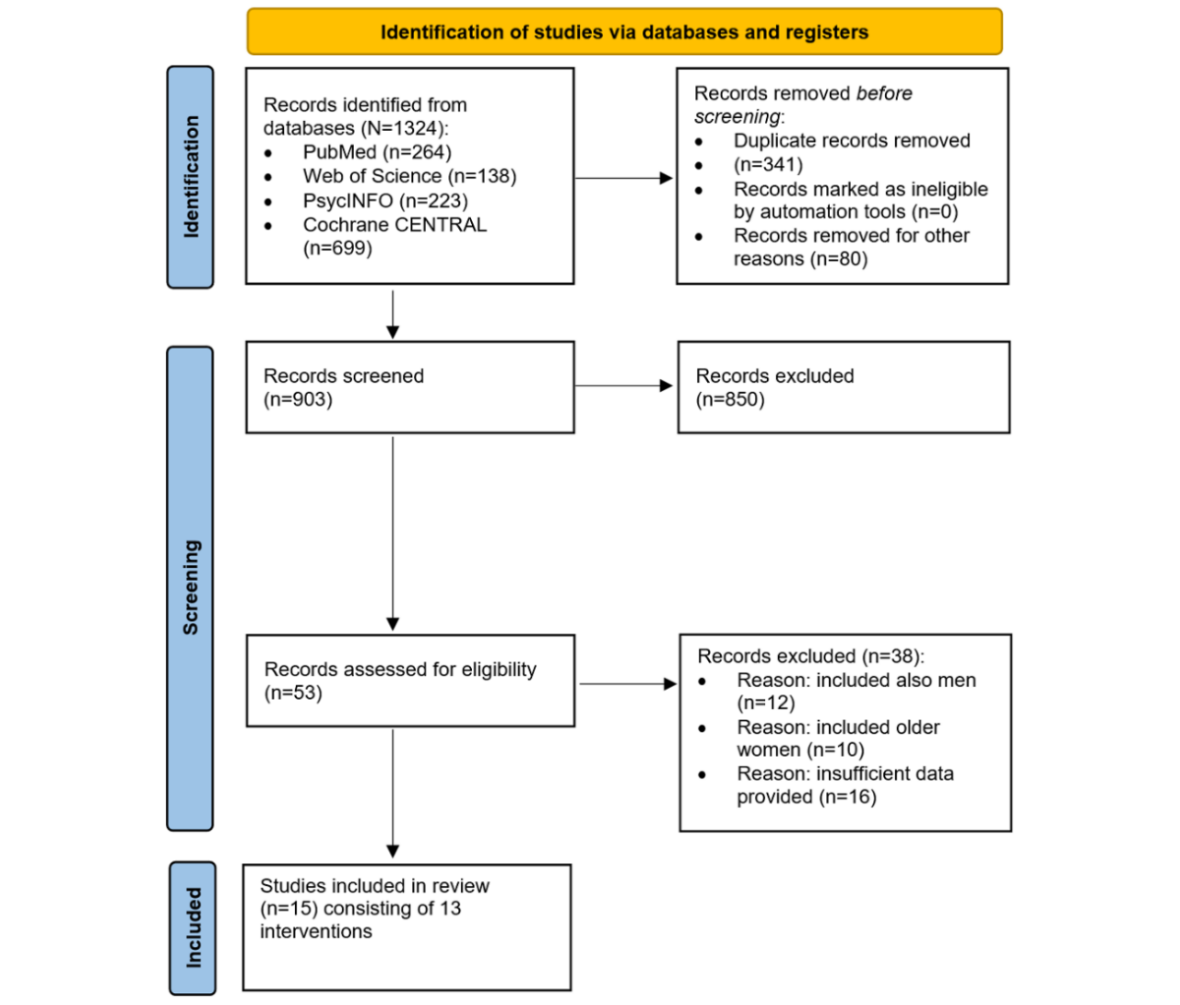
Study selection flow diagram based on the PRISMA (Preferred Reporting Items for Systematic Reviews and Meta-Analyses) statement [55].

Initial searches highlighted 1324 records from databases and included 5 additional records from the *Menopause*, *Climacteric*, and *JMIR* journals. Screening the titles highlighted 25.76% (341/1324) of duplicates and an additional 6.42% (85/1324) of records that were excluded for other reasons. The full-text–reviewed 53 eligible studies provided the remaining 15 (28%) studies (investigating 1661 women) comprising 13 intervention designs (involving 1308 women) that were included in the systematic review. Figure 1 illustrates the study selection process based on the PRISMA flow [55].

### Study Characteristics

Of the 15 studies included, 3 (20%) had weight loss [60-62] as their primary aim. A total of 8 studies focused on improving lifestyle factors, including 2 (13%) on diet [48,63] and 6 (40%) on PA [64-69]—with 1 (7%) study on PA and diet [69] and 1 (7%) study on PA and sleep [68]. A total of 27% (4/15) of the studies focused on improving menopausal symptoms [70-73] such as vasomotor symptoms (eg, hot flushes and night sweats) and bone health [72]. The characteristics of two interventions (Activity and Technology [ACTIVATE] [67,68] and the Women’s Wellness Program [WWP] [69,73]) were each reported in 13% (2/15) of the studies and are described only once (Table 1). Of the 13 interventions, 9 (69%) interventions originated in the United States, 3 (23%) in Australia, and 1 (8%) in South Korea. The length of the studies ranged from 8 weeks to 12 months (median 12 weeks). In 69% (9/13) of the interventions, the participants were overweight or obese and, in 31% (4/13), participants were breast cancer survivors. A total of 7% (1/15) of the studies reported having participants with a high risk of hypertension. White individuals participated in 73% (11/15) of the studies, Asian individuals participated in 20% (3/15) of the studies, and 7% (1/15) of the studies had a mix of White and African American participants.

The average age of the participants was 52.25 (SD 4.79, range 45.7-61.6) years. The inclusion criterion was a minimum of 2 participant groups or arms, with 20% (3/15) of the studies (ie, ACTIVATE [67,68], WWP [69,73], and Striving to be Strong [72]) consisting of 3 arms. The comparison groups were either a control group or groups with reduced BCI frequency (eg, diet tracking with no feedback [48]), technology (eg, no technology [69,73] or no SMS text messages [65]), or PA type (eg, endurance group [61]). Where appropriate, an additional review of the studies was provided in three categories (ie, weight loss, lifestyle, and menopausal symptoms) to allow for a more meaningful comparability of the interventions. The outcomes of the studies provided mixed results, with 33% (5/15) reporting statistically significant differences between the intervention and control groups in the primary measured outcomes. This represented 33.18% (434/1308) of all the intervention participants combined (Table 1). Finally, all studies combined (15/15, 100%) had a mean retention rate of 84% (SD 12.6%, range 59%-100%).

**Table 1:** Characteristics of the studies included in the review (N=15).

Study	Behavior change theory^a^	Intervention									
		Type^b^	Study design	Comparison group	Length	Country	Participant ethnicity	Health risk^c^	Participants, N^d^	Age, mean (SD)	*P* value^e^
Grossman et al [61]	Several BCTs^f^	Weight loss	2-arm feasibility pilot	Endurance group	16 weeks	United States	White	Obesity	11	59.0 (5.33)	No
Hartman et al [62]	SCT^g^	Weight loss	2-arm pilot	Usual care	6 months	United States	White	Breast cancer and overweight or obesity	54	45.7 (4.0)	Yes^h^
Park and Kim [60]	Several BCTs	Weight loss	2-arm quasi-experimental RCT^i^	Control group	12 weeks	South Korea	South Korean	Abdominal obesity	67	51.3 (11.31)	Yes^h^
Cadmus-Bertram et al [64]	CALO-RE^j^ framework	Lifestyle (PA^k^)	2-arm RCT	Control group	16 weeks	United States	White	Overweight or obesity	51	58.6 (6.5)	No
Finkelstein et al [65]	Several BCTs	Lifestyle (PA)	2-arm crossover pilot	No SMS text message group	8 weeks	United States	White and African American	Obesity	27	52.0 (12.0)	Yes^h^
Fukuoka et al [66]	SCT (Bandura) and SCM^l^	Lifestyle (PA)	3-arm parallel RCT	Control group	12 weeks	United States	White	Overweight or obesity	210	52.4 (11.2)	Yes^h^
Lynch et al [67] and Nguyen et al [68]	MI^m^ and several BCTs	Lifestyle (PA and sleep)	2-arm RCT	Control group	12 weeks	Australia	White	Breast cancer and overweight or obesity	83	61.6 (6.4)	Yes^h^ and no
McGuire et al [69] and Anderson et al [73]	SCT (Bandura)	Lifestyle (PA) and menopausal symptoms	3-arm equivalency RCT	No-technology group (group B)	12 weeks	Australia	White	Breast cancer and overweight or obesity	225	50.9 (5.9)	No
Ryan et al [63]	ITHBC^n^	Lifestyle (diet)	2-arm repeated-measure experimental RCT	Usual care	6 months	United States	White	N/A^o^	148	50.11 (5.53)	No
Steinberg et al [48]	Several BCTs	Lifestyle (diet)	2-arm feasibility RCT	Control group (active)	12 weeks	Australia	White	Hypertension	59	49.9 (11.9)	No
Im et al [70]	SET^p^ (Bandura)	Menopausal symptoms	2-arm repeated-measure RCT	Control group	12 weeks	United States	Asian American	N/A	29	45.7 (4.0)	No
Im et al [71]	SET (Bandura)	Menopausal symptoms	2-arm repeated-measure RCT	Control group	12 weeks	United States	Asian American	Breast cancer	91	51.3 (11.31)	No
Ryan et al [72]	IFSMT^q^	Menopausal symptoms	3-arm prospective repeated-measure longitudinal RCT	Waitlist	12 months	United States	White	Overweight	260	50.57 (5.19)	No

^a^Behavior change theory consisted of (1) several behavior change techniques; (2) the Social Cognitive Theory; (3) the Coventry, Aberdeen, and London-Refined framework; (4) the Self-Efficacy Theory; (5) the Integrated Theory of Health Behavior Change; and (6) the Individual and Family Self-Management Theory.

^b^Intervention outcome types included (1) weight loss, (2) lifestyle (physical activity), (3) lifestyle (diet), (4) lifestyle (sleep), and (5) menopausal symptoms.

^c^Health risks included (1) obesity, (2) breast cancer, (3) overweight or obesity, (4) hypertension, (5) overweight, and (6) abdominal obesity.

^d^Number of participants in the intervention.

^e^Statistically significant between-group differences.

^f^BCT: behavior change technique.

^g^SCT: Social Cognitive Theory.

^h^*P*<.05.

^i^RCT: randomized controlled trial.

^j^CALO-RE: Coventry, Aberdeen, and London-Refined.

^k^PA: physical activity.

^l^SCM: Stages of Change Model.

^m^MI: Motivational Interviewing.

^n^ITHBC: Integrated Theory of Health Behavior Change.

^o^N/A: not applicable.

^p^SET: Self-efficacy Theory.

^q^IFSMT: Individual and Family Self-Management Theory.

### BCTs and Categories Used

Overall, the 13 interventions used a range of 6 to 21 BCTs (mean 13.0, SD 4.3, median 13), representing 6% to 23% (median 14%) of the available 93 BCTs from the BCTTv1 [53] (Table 2). Nine BCTs (ie, “instructions on behaviour,” “feedback on behavior,” “habit formation,” “behavioural practice/rehearsal,” “action planning,” “prompts/cues,” “goal setting (behaviour),” “self-monitoring of behaviour,” and “graded tasks”) were used by more than half (7/13, 54%) of the interventions. In addition, two BCTs (ie, “instructions on behaviour” and “feedback on behaviour”) were used by 92% (12/13) and 85% (11/13) of the interventions, respectively (Multimedia Appendix 2 [48,60-73]). Examples of “instructions on behaviour” included providing participants with DVD-guided training instructions, coaching calls that included instructions on meal planning and increasing vegetable intake, or daily video clips about healthy diet and weight maintenance. Examples of “feedback on behaviour” included receiving individualized weekly feedback on activity recording and adherence to the dietary program and, upon recording food intake, receiving immediate feedback on how many calories are left until the participant’s daily goal is reached. The next two frequently implemented BCTs—“habit formation” and “behavioral practice/rehearsal”—were each implemented in 77% (10/13) of the interventions. Examples of “habit formation” included researchers sending 3 messages per week or reinforcing content through daily messages and videos. “Behavioural practice/rehearsal” was implemented by providing weekly activity planning with participants to identify and reflect on their barriers to exercising behavior change through journal activities, reflections, and discussion. Finally, approximately 69% (9/13) of the interventions used the “action planning” or “prompts/cues” BCTs.

There was no single cluster of BCTs from which all 13 interventions selected at least one BCT (ie, no behavior change category reached 100%), and only 44% (7/16) of the behavior change categories were used by more than half (7/13, 54%) of the interventions (Table 3). The most frequently used seven categories or clusters of BCTs—from which more than half of the interventions (7/13, 54%) used at least one BCT—were “shaping knowledge,” “repetition and substitution,” “feedback and monitoring,” “goals and planning,” “social support,” “associations,” and “antecedents.” Furthermore, 54% (7/13) of the interventions used at least one BCT in 16 of the available behavior change categories, whereas four clusters of BCTs (ie, “regulation,” “identity,” “self-belief,” and “covert learning”) were not used by any study (Table 3).

**Table 2:** Number of behavior change techniques (BCTs) and BCT categories used across all studies.

	Grossman et al [61]	Hartman et al [62]	Park and Kim [60]	Cadmus-Bertram et al [64]	Finkelstein et al [65]	Fukuoka et al [66]	Lynch et al [67] and Nguyen et al [68]	Anderson et al [73] and McGuire et al [69]	Ryan et al [63]	Steinberg et al [48]	Im et al [70]	Im et al [71]	Ryan et al [72]	BCTs per category, n (%)^a^	Mean (SD)^b^
Goals and planning (9 BCTs)	4	5	1	3	—	4	4	3	5	2	—^c^	—	3	34 (20)	2.62 (1.85)
Feedback and monitoring (7 BCTs)	4	2	4	3	2	2	2	1	2	4	—	—	2	28 (17)	2.15 (1.34)
Social support (3 BCTs)	1	—	—	—	—	1	1	1	1	2	2	1	1	11 (7)	0.85 (0.69)
Shaping knowledge (4 BCTs)	1	1	1	1	1	1		1	1	1	1	1	1	12 (7)	0.92 (0.28)
Natural consequences (6 BCTs)	—	—	—	—	—	1	1	1	—	—	1	1	—	5 (3)	0.38 (0.51)
Comparison of behavior (3 BCTs)	1	—	—	—	—	—	—	1	1	1	—	—	1	5 (3)	0.38 (0.51)
Associations (8 BCTs)	—	2	1	—	1	1	2	1	1	2	—	1	—	12 (7)	0.92 (0.76)
Repetition and substitution (7 BCTs)	3	1	5	1	2	3	2	3	3	—	2	2	3	30 (18)	2.31 (1.25)
Comparison of outcomes (3 BCTs)	1	1	1	—	—	—	—	1	1	—	—	1	—	6 (4)	0.46 (0.52)
Reward and threat (11 BCTs)	2	—	—	2	—	2	—	—	2	2	—	—	3	13 (8)	1.00 (1.15)
Regulation (4 BCTs)	—	—	—	—	—	—	—	—	—	—	—	—	—	—	—
Antecedents (6 BCTs)	3	1	1	1	1	1	1	—	—	—	—	—	1	10 (6)	0.77 (0.83)
Identity (5 BCTs)	—	—	—	—	—	—	—	—	—	—	—	—	—		
Scheduled consequences (10 BCTs)	1	—	—	1	—	—	—	—	—	—	—	—	1	3 (2)	0.23 (0.44)
Self-belief (4 BCTs)	—	—	—	—	—	—	—	—	—	—	—	—	—	—	—
Covert learning (3 BCTs)	—	—	—	—	—	—	—	—	—	—	—	—	—	—	—
BCTs per study, n (%)^d^	21 (12)	13 (8)	14 (8)	12 (7)	7 (4)	16 (9)	13 (8)	13 (8)	17 (10)	14 (8)	6 (4)	7 (4)	16 (9)	169 (100)^e^	13.00 (4.30)

^a^The total number of BCTs used across all 13 interventions for each behavior change category. In the table, the number of BCTs in each study is represented by a number. Studies with absent BCTs in each behavior change category are marked with —.

^b^The average number of BCTs used in each behavior change category across all 13 interventions and the SD of the mean number of BCTs used in each behavior change category.

^c^Not applicable.

^d^The total number of BCTs used within each intervention and the percentage of BCTs each study used from the total number of BCTs across all studies.

^e^The sum of the total number of BCTs used across all 16 behavior change categories and all 13 interventions.

**Table 3:** Behavior change technique (BCT) category results by study type (ie, all, weight loss, lifestyle, and menopause symptoms) where at least one BCT was used in each BCT category.

BCT categories	Scoring for all studies with ≥1 BCT, %	Scoring for weight loss studies with ≥1 BCT, %	Scoring for lifestyle studies with ≥1 BCT, %	Scoring for menopause symptom studies with ≥1 BCT, %
Goals and planning	77	100	88	50
Feedback and monitoring	85	100	100	50
Social support	69	33	75	100
Shaping knowledge	92	100	75	100
Natural consequences	38	0	50	75
Comparison of behavior	38	33	38	50
Associations	69	67	88	50
Repetition and substitution	92	100	88	100
Comparison of outcomes	46	100	25	50
Reward and threat	46	33	50	25
Regulation	0	0	0	0
Antecedents	62	100	63	25
Identity	0	0	0	0
Scheduled consequences	20	33	13	25
Self-belief	0	0	0	0
Covert learning	0	0	0	0

### BCW Mapping

#### Overview

A total of 89% (8/9) of the BCW intervention functions were used in the interventions. The most commonly used intervention functions were “enablement” (60/169, 35.5%), “training” (32/169, 18.9%), “persuasion” (23/169, 13.6%), and “education” (18/169, 10.7%). “Incentivisation” (14/169, 8.3%), “environmental restructuring” (7/169, 4.1%), and “coercion” (2/169, 1.2%) were used to a smaller degree. “Restriction” and “modelling” were not used by any intervention. The COM-B model at the core of the BCW showed that 50% (49/98) of the intervention strategies focused on increasing “capability,” 42% (41/98) focused on increasing “motivation,” and 8% (8/98) focused on providing “opportunity.” Furthermore, a breakdown of the “capability” component suggests that 42% (41/98) were linked to “psychological capability” and 8% (8/98) were linked to “physical capability.” The “opportunity” components show that 3% (3/98) and 5% (5/98) were related to “social” and “physical opportunity,” respectively. Finally, expanding the “motivation” component suggests that 35% (34/98) and 7% (7/98) were linked to “reflective” and “automatic motivation,” respectively (Multimedia Appendix 3). The TDF framework components within the COM-B model of the BCW indicate that the mechanism of action for the BCTs used most frequently was “behavioural regulation” (15/98, 15%), primarily in the “goals and planning” (6/15, 40%; eg, instructions to rotate through 5 different workouts before progression and setting an initial weight loss goal) and “repetition and substitution” (6/15, 40%; eg, progression from 60 minutes of exercise in week 1 to 250 minutes of exercise in week 15 and sending 3 SMS text messages per week) behavior change categories. Additional TDF domains used most frequently within the “repetition and substitution” behavior change category were increasing “knowledge,” “skills,” and “cognitive and interpersonal skills.” “Motivation” was increased primarily through “beliefs about capabilities” (eg, providing daily feedback on steps walked to help the participants monitor and adjust goals) and “professional or social role and identity” (eg, providing monthly face-to-face group meetings and access to a web-based forum to discuss experiences and receive individual and group coaching support). “Opportunity” was increased primarily by “physical environmental restructuring” such as providing instructions on modifications to food and exercise environments (eg, stocking the kitchen with healthy foods and packing exercise clothes ahead of time; Multimedia Appendix 3).

#### Weight-Loss Intervention Group

A total of 20% (3/15) of the studies aimed to induce weight loss in midlife women [60-62], with all using exercise and diet interventions. The mean frequency of BCTs across the 3 weight loss studies was 16 (SD 4.36, range 13-21). The BCT categories “goals and planning,” “feedback and monitoring,” and “repetition and substitution” were used most frequently, with 10, 10, and 9 BCTs, respectively. The BCW mapping suggests that increasing “capability” was implemented in 55% (42/77) of the behavior change interactions, followed by increasing “motivation” in 36% (28/77) of the interactions and increasing “opportunity” in 9% (7/77) of the interactions. Furthermore, the “psychological capability” TDF domain was used the most frequently, specifically through “behavioural regulation.”

#### Lifestyle Intervention Group

Of the 8 studies included in the lifestyle group of interventions, 3 (38%) aimed to improve PA through a PA program [64-66]. A total of 25% (2/8) of the studies aimed to improve well-being through diet [48,63]. The McGuire et al [69] study of WWP trial aimed to improve PA through diet and exercise. In total, 25% (2/8) of the studies were from the ACTIVATE trial; one aimed to improve sleep by improving PA [68] and another to improve PA through exercise and coaching [67]. The mean frequency of BCTs across the lifestyle studies was 13.13 (SD 3.00, range 7-17). The BCT categories “goals and planning,” “feedback and monitoring,” and “repetition and substitution” were used most frequently, with 25, 18, and 16 BCTs, respectively. The BCW mapping shows that motivation was used in 45% (31/69) of BCTs, similar to capability at 46% (32/69) followed by increasing “opportunity” at 9% (6/69). The “Psychological capability” TDF domain was used the most frequently, with “behavioural regulation” followed by increasing “knowledge” (eg, self-monitoring food intake and PA) and “building competencies” (eg, encouragement to enter consumed foods in real time and receiving immediate feedback on goal progression).

#### Menopause Symptom Intervention Group

A total of 27% (4/15) of the studies were included in the menopause symptom interventions group [70-73] among which one study [73] measured menopausal symptoms such as depression, anxiety, and somatic and vasomotor symptoms using the Greene Climacteric Scale [76]. The intervention was based on promoting healthy lifestyle behaviors, emphasizing a healthy diet and regular PA. Im et al [70] aimed to improve menopausal symptoms by emphasizing PA. By contrast, the aim of the study by Im et al [71] was to decrease menopausal symptoms through education and coaching. Ryan et al [72] measured bone mineral density among three groups (2 intervention and 1 control). The study used an ecological momentary assessment software to encourage the participants to increase their calcium intake, PA, balance, and strength [72]. The average frequency of BCTs across the 4 menopause symptom studies was 10.50 (SD 4.80, range 6-16). The BCT categories “repetition and substitution,” “goals and planning,” “social support,” and “shaping knowledge” were used most frequently, with 10, 6, 5, and 4 BCTs, respectively. The BCW mapping shows that “motivation” was used in 44% (24/55) of BCTs, similar to “capability” at 47% (26/55) followed by increasing “opportunity” at 9% (5/55). The BCW mapping suggests that the “psychological capability” TDF domain was used the most frequently, specifically through “behavioural regulation.”

### Extent of Theory Use

The overall mean total use of theory score (based on the TCS) for all interventions was 8/23 (SD 3.87, range 4-15), which represents a weak level (score 8-15; Multimedia Appendix 4 [48,60-73]). Individual interventions were scored, with 62% (8/13) categorized as weak (score 0-7) and the remaining 38% (5/13) scoring moderate levels (score 8-15). No study achieved a strong score (score 16-23). Of the 13 interventions, 7 (54%) explicitly reported that they were based on theory (item 5; Multimedia Appendix 4). Of these 13 interventions, 7 (54%) were based on a single theory (item 3), none reported using theory to recruit study participants (item 4), and 3 (23%) reported using theory to tailor BCTs to recipients (item 6). Of these 13 interventions, none explicitly reported links between all BCTs within the intervention and the targeted theoretical constructs (item 7), whereas 4 (31%) reported targeting all the constructs within a specified theory with specific BCTs (item 10). A total of 62% (8/13) of the interventions reported measuring theoretical constructs after the intervention, and 62% (8/13) measured constructs both before and after the intervention (item 12). However, only 62% (8/13) of interventions reported statistically significant mediated effects (item 16d). Only 23% (3/13) of the interventions reported suggestions for theoretical refinement based on their findings (item 19). The review of the 6 TCS categories suggests that 77% (10/13; mean 3/7, SD 1.25) of the interventions stated or suggested rather than demonstrated theoretical base (being based on theory; category 1). All 13 interventions targeted theoretical constructs that predicted behavior (category 2; mean 2.69/7, SD 1.84).

### Behavior Change Theories Used

Although all 13 interventions mentioned behavior change, a specific behavior change theory was mentioned in 69% (9/13) of the interventions. The most frequently used behavior change theories included the Self-Efficacy Theory (SET) and Social Cognitive Theory (SCT), each being implemented in the design of 15% (2/13) of the interventions. The Stages of Change Model; Individual and Family Self-Management Theory; Integrated Theory of Health Behavior Change; Motivational Interviewing; and Coventry, Aberdeen, and London-Revised [77] framework were each used in 7% (1/15) of the studies. The remaining 27% (4/15) of the studies that mentioned behavior change reported using several BCTs (Table 1).

### Modes of Delivery Used in the Studies

#### Overview

The studies used a combination of technological and nontechnological components. Websites were used in 69% (9/13) of the interventions, and phone or SMS text messages were used in 62% (8/13) of the interventions, followed by wearables, which were used in 54% (7/13) of the interventions (Multimedia Appendix 5 [48,60-73]). Apps, email, electronic documents, and ecological momentary assessment were used in 46% (6/13), 23% (3/13), 15% (2/13), and 8% (1/13) of the interventions, respectively. In addition, 65% (46/71) of the technology interactions with the participants in all studies were provided in a passive manner without the participants’ active involvement (eg, providing health and lifestyle information such as recipes, tips, and frequently asked questions). By contrast, 35% (25/71) of the interactions were provided in an action-based manner. Evaluation of technological features provided in the interventions showed that the top 3 interactions included health or lifestyle information, which was provided in 24% (16/68) of all the interactions; activity tracking, which was provided in 19% (13/68) of the interactions; and health or lifestyle lessons, which were provided in 10% (7/68) of the interactions. Furthermore, social media and support provided 7% (5/68) of the interactions; web-based health coaching provided 3% (2/68) of the interactions; and barrier tracking, activity tracking, and health education each provided 3% (2/68) of the interactions. Other technological features such as reminders or prompts, social support, health information, health feedback, health activity, social support, practical support, diet tracking, and follow-up each provided 1% (1/68) of the interactions. Nontechnological components such as face-to-face interactions and providing hard-copy intervention material were used by 38% (5/13) and 46% (6/13) of the interventions, respectively.

#### Weight-Loss Intervention Group

In addition to technical components, 13% (2/15) of the studies [61,62] also used nontechnological components such as face-to-face meetings and providing a hard copy of the intervention material. Of the technical components, 67% (8/12) were passive, whereas 33% (4/12) were action based.

#### Lifestyle Intervention Group

Of the technical components, 69% (24/35) were passive, whereas 31% (11/35) were action based. Most studies (6/8, 75%) also used nontechnical components such as face-to-face meetings and hard-copy study documentation.

#### Menopause Symptom Intervention Group

Of the technical components, 62% (16/26) were passive, whereas 38% (10/26) were action based. Only the WWP study by Anderson et al [73] used nontechnical components such as face-to-face meetings and a hard copy of the program book.

### Fidelity of the Studies

Of the 13 interventions, 8 (62%) included an assessment of all 5 domains (Multimedia Appendix 6 [48,60-73]). The greatest average proportion of adherence to treatment fidelity across all 13 interventions was in the “Enactment” domain at 50% (0.50). The lowest mean proportion of adherence to strategies was found in the “Receipt” domain, where, on average, only 26% (0.26, SD 0.25) of strategies were reported among the studies. Finally, the mean proportion of adherence to strategies in the “Treatment,” “Training,” and “Delivery” domains was 45% (0.45, SD 0.18), 34% (0.34, SD 0.22), and 32% (0.32, SD 0.14), respectively. The mean proportion of adherence to treatment fidelity strategies included across all 5 domains for all studies was 0.39 (SD 0.14, median 0.41). On the basis of the fidelity scoring by Borrelli [74], where 50% constitutes low-fidelity scoring, 85% (11/13) of the interventions scored a low treatment fidelity across all 5 domains. In total, 13% (2/15) of the studies, both by Ryan et al [63,72], scored >0.50 in the medium treatment fidelity range (ie, 0.51 to 0.79), with 0.62 and 0.59 treatment fidelity. For details of scoring for each component of the treatment fidelity domain, see Multimedia Appendix 6.

## Discussion

### Principal Findings

This review systematically reviewed 13 interventions that aimed to improve weight loss (3/15, 20%), lifestyle (8/15, 53%), and menopause symptoms (4/15, 27%) through DHIs in midlife women. Six BCTs (ie, “Feedback on behaviour,” “Prompts/cues,” “Action planning,” “Instructions on behaviour,” “Behavioural practice/rehearsal,” and “Habit formation”) were used in at least 80% (4/5) from the studies that showed significant between-group differences in main outcomes. This group of studies used an average of 12.6 BCTs (range 7-16, median 13), representing 13.98% (169/1209) of all BCTs available from the BCTTv1 taxonomy. The most frequently used six clusters of BCTs (ie, “Feedback and monitoring,” “Associations,” “Repetition and substitution,” “Antecedents,” “Shaping knowledge,” and “Goals and planning”) were used by >80% (4/5) of the studies. Four clusters of BCTs (ie, “Social support,” “Natural consequences,” “Comparison of outcomes,” and “Reward and threat”) were used by only 20% (1/5) to 40% (2/5) of the studies. Six other clusters (ie, “Regulation,” “Identity,” “Self-belief,” “Covert learning,” “Comparison of behaviour,” and “Scheduled consequences”) were not used, which may indicate that the BCTs within these clusters were unexplored or potentially found inappropriate for these interventions. Although the findings indicate which BCTs are used more frequently in health-enhancing DHIs with midlife women, the high level of heterogeneity in the design of the interventions and selection of specific BCTs suggests that the designs of these interventions cannot be generalized across various contexts. DHIs should consider the unique experiences and needs of women in midlife, including marginalized women, to improve their sociodemographic diversity.

In this review, 78% (7/9) of BCW intervention functions were identified, with a strong emphasis on “enablement” (eg, encouragement to set an initial weight loss goal and self-monitoring food intake and PA) by increasing capability beyond education and training. “Training” and “persuasion” were also commonly used, whereas “restriction” and “modelling” were not used at all. In a nondigital lifestyle BCI (involving adult men and women), 5 (56%; 5/9) of the intervention functions were used (ie, “enablement,” “training,” “persuasion,” “restriction,” and “education”), whereas “incentivization,” “coercion,” and “modelling” were not used and were found to be inappropriate in the context of the intervention [78]. In another nondigital lifestyle behavior change review, education (eg, nutritional label reading and a resistance training booklet for exercise) was the most commonly used intervention function, being present in 81% of the interventions [79]. “Enablement” (eg, self-management techniques to foster self-efficacy and arranging support from friends and family) and “training” (eg, home-based exercise training, guided exercise training, and hands-on cooking classes) were also emphasized, whereas “coercion” and “restriction” were not used in any of the interventions [79]. Overall, “education,” “enablement,” and “training” were used commonly across digital and nondigital intervention types, whereas “coercion” or “restriction” were used less commonly.

When comparing digital and traditional face-to-face (ie, nondigital) lifestyle health-enhancing interventions, there are apparent commonalities and differences in the BCT clusters typically used within interventions. Previous reviews have highlighted that only a fraction (34%; 32/93) of the BCTs were used across all interventions, with the “Feedback and monitoring” and “Goals and Planning” BCT clusters used more commonly in traditional lifestyle interventions [79,80], which aligns with what was observed in this review. Contrary to previous reports on traditional lifestyle interventions, this study demonstrated that DHIs in midlife women were more likely to use “Repetition and substitution” (ie, habit formation) techniques [79,80]. This difference may be due to the just-in-time nature of digital technologies, which allows for the implementation of behaviors that may emerge rapidly, unexpectedly, and ecologically and that are usually less accessible with in-person approaches [81].

In other DHIs, certain BCTs were found to be more frequently applied on particular technological platforms. For example, the most frequently used BCTs in lifestyle interventions using mobile apps were “feedback on behaviour” (84%; 26/31), “self-monitoring of behaviour” (77%; 24/31), and “goal setting” (61%; 19/31) [82]. Although these BCT features were apparent in the mobile app–based interventions included in this review, they were not universally applied. Equally, digital PA BCIs used primarily a combination of “goal setting,” “self-monitoring,” and “motivation,” whereas digital healthy eating interventions primarily targeted “self-monitoring,” “goal setting,” and “feedback on behaviour” [82]. Similarly, in this review, “feedback on behaviour” (11/13, 85%), “goal setting” (8/13, 62%), and “self-monitoring” (8/13, 62%) were in the top 10 BCTs used across all technological platforms in all studies. In gamification platforms, for example, the most frequently used BCTs were “education” and “reward” as these are important features of gamification [44]. This highlights that almost all the key BCTs can be used on a mobile platform, most likely because of the flexibility and accessibility of this technology [44]; therefore, interventions can be easily tailored to the context in which they are being applied. Health interventions for midlife women must be cognizant of the multiple co-occurring stressors that are born from psychosocial and physiological transitions during this period [83]. Interestingly, DHIs have been suggested to be most effective in facilitating problem-solving, encouraging self-efficacy, and reducing the impact of stress associated with behavior change [24].

This review highlighted a varied use of theories of behavior and behavior change to design DHIs, with SET and SCT being most commonly used in DHI research to date. Interventions informed by these theories can effectively enhance PA in midlife women [18,84]; however, the application of theory to BCT intervention functions has been poorly reported or used [54,57]. In this review, although 69% (9/13) of the interventions mentioned behavioral theory, more than half (8/13, 62%) had weak scores (based on TCS) in applying theory to the intervention. In cases where the intervention was reportedly based on theory (ie, SCT, SET, and the Stages of Change Model), none of the studies in this review explicitly linked all theoretical constructs with BCTs and vice versa. As such, having a theoretical understanding of behavior change is necessary to maximize the potential efficacy of interventions [85].

A critical component of intervention delivery is establishing theoretical fidelity and ensuring that a theory is adequately reflected in the intervention’s design and implementation [74]. The overall poor reporting of treatment fidelity in this review (with only 2/15, 13% of the interventions reporting medium treatment fidelity [63,72]) is similar to other reviews that considered fidelity [41,74,86]. Although 69% (9/13) of the interventions in this review mentioned a theory, the treatment design domain achieved only a low mean proportion of treatment fidelity. When intervention effects are not significant, treatment fidelity helps understand whether this is due to the omission or addition of active or inactive components or whether it is due to an ineffective treatment [74]. However, it is important to highlight that a lack of effect may reflect implementation failure rather than genuine ineffectiveness and, through the evaluation process, implementation problems can be identified [20]. Although this review combined treatment fidelity for studies that did and did not achieve statistically significant group differences, an accurate estimate of the relationship between theory use and intervention effectiveness can only be obtained from studies that reach high fidelity of delivery [87]. DHIs incorporating behavior change theory offer a unique opportunity to refine and strengthen the theory. Unfortunately, none of the studies in this review reported refining or developing a theory to strengthen intervention effectiveness. Moller et al [88] explored potential improvements for applying behavior change theories in the context of digital health and suggested that digital technologies may potentially provide high fidelity of delivery owing to their ability to measure engagement levels objectively. Furthermore, digital technologies can also access large data sets generated by ecologically valid measures of behavior, emotion, physiology, and thinking in real time and everyday contexts [88]. Therefore, considering treatment fidelity in DHIs is essential to estimate the confidence with which intervention effects can be attributed to BCTs.

Although extending the interpretation of the findings to the effectiveness of certain BCTs was outside the scope of this review, it should be noted that identifying effective BCTs and a combination of BCTs for a given behavior in a given context presents a major challenge [89]. Research evaluating the effectiveness of BCTs and BCT combinations uses a range of observational and experimental methods, each with strengths and limitations [89]. For example, van Rhoon et al [90] drew conclusions on the effectiveness of specific numbers and types of BCTs in weight loss DHIs based on the BCTs that were present in interventions producing clinically significant weight loss outcomes. However, this method led to the inability to identify the mechanisms by which the BCTs and digital features influenced the target behavior. In addition, this method runs the risk of including BCTs that do not add to effectiveness but happened to be included in the effective interventions [89]. Furthermore, although other evaluation methods such as meta-analyses can provide generalizable conclusions [89], poor quality in intervention description and high heterogeneity in the designs may not allow for statistical analysis of the effectiveness of individual BCTs or a combination of BCTs on the intervention outcomes [21,44,78,91,92]. Making confident estimates of the effectiveness of BCTs and BCT combinations for a given behavior delivered in a particular way in a given setting to a given target population requires synthesis of information from diverse sources [89]. This challenge provides an opportunity for future research to develop a strategy that systematically combines the strengths of the different methods and that links these constructs in an ontology of BCIs [89].

The outcomes of this review suggest that the effects of BCTs on behavior are difficult to determine because of high heterogeneity in the designs of the interventions and low level of treatment fidelity and theoretical grounding. It is also important to note that, although the BCW provides a systematic and theory-guided method for identifying components of interventions and types of interventions that are expected to be effective, it does not provide a detailed blueprint for the design of specific BCIs [19]. Therefore, the BCW framework should be applied with a level of flexibility, as acknowledged by its authors [78]. Furthermore, although theory-based intervention design is critical for intervention effectiveness [20], the involvement of key stakeholders in the development process of interventions through coproduction increases the likelihood of the intervention meeting user needs and their implementation [20,93]. Although most of the participants in the studies in this review were White individuals (9/13, 69% of the interventions), research shows that menopausal symptom experiences vary among women with different sociodemographic characteristics, including ethnicity, income, and education [94-96]. The lack of diversity in the sociodemographic characteristics of the studies and the apparent lack of evidence on how to culturally adapt DHIs provide an opportunity to explore these topics in future research. In addition, co-designing theory- and evidence-based interventions with Patient and Public Involvement in all stages of the design process [92,97-99] would be beneficial to ensure that digital health lifestyle BCIs for midlife women are acceptable, feasible, and more effective. To date, there has been no such study undertaken and, therefore, this represents an opportunity for researchers to advance the field by improving both the quality and replicability of such interventions.

### Strengths and Limitations

The process of identification of the BCTs requires classification and coding of intervention descriptions [77] using the BCTTv1 [53] for each study. The level of detail necessary for BCT coding was limited in the studies. As such, this review contains a possible subjectivity limitation in categorizing, reviewing, and mapping behavior change theories and BCTs. To mitigate this limitation, two researchers (HS and SD) interpreted and coded the BCTs to reduce any bias (also acknowledged in other research [44]). Similarly, to reduce bias in interpreting and coding, the TCS items (also acknowledged in other research [41]) were completed by 2 researchers. Improving the description of the intervention design and delivery is essential for improving BCT coding to better facilitate scientific evaluation and translational processes in future studies.

Furthermore, the number of health-promoting studies designed specifically for midlife women is limited. This review contains a small number of studies with limited sociodemographic (ie, the participants in 9/13, 69% of the interventions were White individuals) and socioeconomic (ie, all the included studies came from high-income countries) backgrounds and attempts to assess the quality of evidence that may not be generalizable to all digital health BCIs with midlife women. Future research should consider the unique needs of women of diverse sociodemographic and socioeconomic backgrounds in their intervention designs to make their findings applicable to more women.

### Conclusions

This review identified studies aiming to promote lifestyle improvements in midlife women using digital technology and assessed their designs through the application of the BCW framework. The assessment identified gaps in the process of designing digital health BCIs. The studies obtained weak to moderate scores in their theoretical grounding, and their description of intervention components, intervention functions, and BCTs was also weak. The low level of treatment fidelity suggests that the interventions may not have delivered what the researchers intended to deliver (also acknowledged elsewhere [41]) and that the interventions may not be replicable. This suggests, as also highlighted by Michie et al [89], that there is a need for better tools and intervention design guidelines to facilitate better selection and use of behavioral theories. Although the findings indicate which BCTs are used more frequently in specific groups of interventions, the high level of heterogeneity in the design of the interventions and selection of specific BCTs suggests that the designs of these interventions cannot be generalized across different contexts. Instead, applying the principles underlying the design of these groups of interventions through systematically co-designing theory- and evidence-based interventions with midlife women may be more efficacious. Further research is needed to validate such intervention designs and their application in feasibility and acceptability studies. A closer collaboration between behavioral science and solution design is needed to bridge this gap and increase the effectiveness of digital health behavior change technologies.

## Appendix

Multimedia Appendix 1 Quality assessment results.

Multimedia Appendix 2 Behavior change theory mappings across all studies.

Multimedia Appendix 3 Behavior change wheel mapping of all studies.

Multimedia Appendix 4 Theory Coding Scheme scoring across all studies.

Multimedia Appendix 5 Technological components used in each study.

Multimedia Appendix 6 Treatment fidelity of the studies.

## Abbreviations

ACTIVATE: Activity and Technology intervention
BCI: behavior change intervention
BCT: behavior change technique
BCTTv1: Behavior Change Technique Taxonomy v1
BCW: behavior change wheel
COM-B: capability, opportunity, and motivation-behavior
DHI: digital health intervention
PA: physical activity
PRISMA: Preferred Reporting Items for Systematic Reviews and Meta-Analyses
SCT: Social Cognitive Theory
SET: Self-Efficacy Theory
TCS: Theory Coding Scheme
TDF: Theoretical Domains Framework
WWP: Women’s Wellness Program
