# Supplementary material for: Behavior Change Techniques in Digital Health Interventions for Midlife Women: Systematic Review
Source: JMIR Mhealth Uhealth. 2022 Nov 9;10(11):e37234. doi: 10.2196/37234 (PMC9685514; doi:10.2196/37234)
Supplement: Multimedia Appendix 1 [file mhealth_v10i11e37234_app1.pdf]

This is a Multimedia Appendix to a full manuscript published in the JMIR mHealth and uHealth. For full copyright and citation information see http://dx.doi.org/10.2196/jmir.37234

The quality assessment was completed using the Physiotherapy Evidence Database (PEDro) scale [58] and the Cochrane risk-of-bias tool for randomised trials (RoB 2) [59] was used to assess the risk of bias in randomised trials. The majority (12/13, 92%) of the studies met the modified 6-point PEDro scale at 80-100% (5 points out of 6 points) (**Figure 1**).

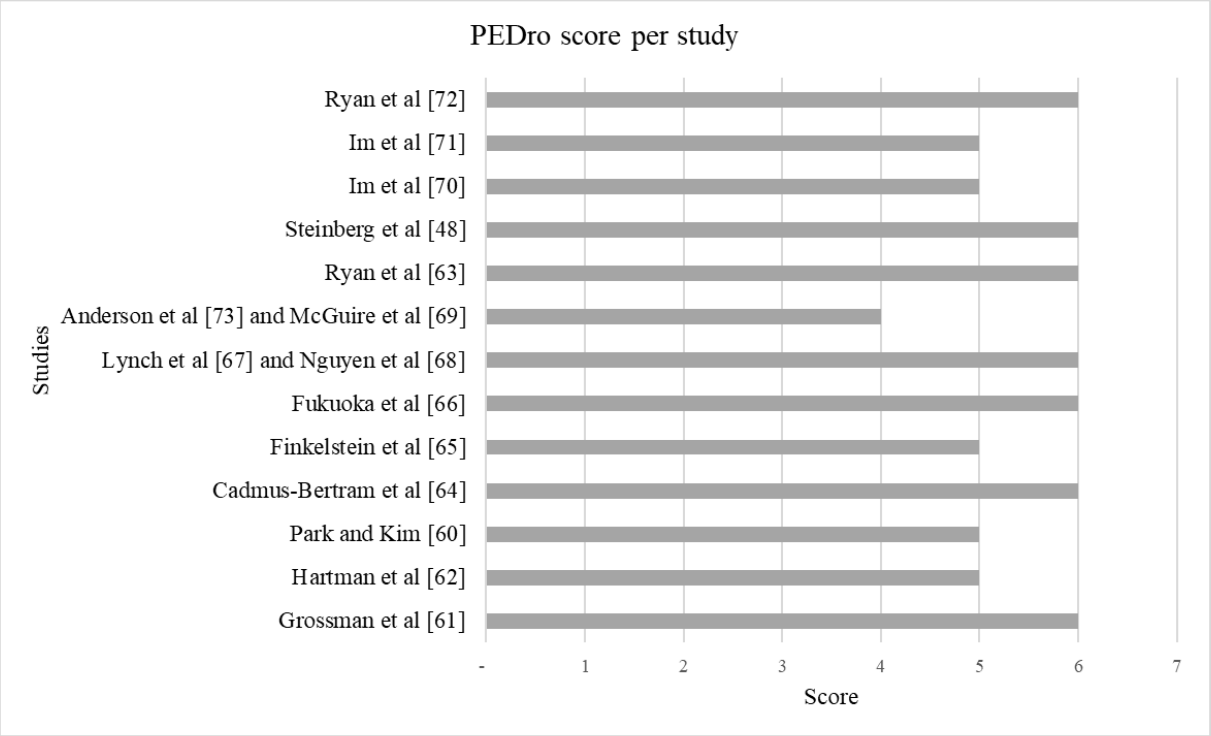

Figure 1. PEDro scale analysis of the included studies

The Cochrane's Risk Bias Analysis indicates that the overall risk of bias was low in 77% (10/13) of the studies, with some concerns in 23% (3/13) of the studies, primarily in the randomisation and deviations from the intended intervention (**Figure 2**).

Study	D1	D2	D3	D4	D5	Overall
Grossman et al [61]	+	!	+	+	+	!
Hartman et al [62]	-	+	+	+	+	!
Park and Kim [60]	+	+	+	+	+	+
Cadmus-Bertram et al [64]	+	+	+	+	+	+
Finkelstein et al [65]	+	+	+	+	+	+
Fukuoka et al [66]	+	+	+	+	+	+
Lynch et al [67] and Nguyen et al [68]	+	+	+	+	+	+
Anderson et al [73] and McGuire et al [69]	-	!	!	+	+	!
Ryan et al [63]	+	+	+	+	+	+
Steinberg et al [48]	+	+	+	+	+	+
Im et al [70]	+	!	+	+	+	+
Im et al [71]	+	+	+	+	+	+
Ryan et al [72]	+	+	+	+	+	+

Legend:

D1 Randomization process
D2 Deviations from the intended interventions
D3 Missing outcome data
D4 Measurement of the outcome
D5 Selection of the reported result

+ Low risk

! Some concerns

- High risk

Figure 2. Cochrane risk of bias analysis of the included studies using Rob2 tool
