# Supplementary material for: Behavior Change Techniques in Digital Health Interventions for Midlife Women: Systematic Review
Source: JMIR Mhealth Uhealth. 2022 Nov 9;10(11):e37234. doi: 10.2196/37234 (PMC9685514; doi:10.2196/37234)
Supplement: Multimedia Appendix 2 [file mhealth_v10i11e37234_app2.pdf]

This is a Multimedia Appendix to a full manuscript published in the JMIR mHealth and uHealth. For full copyright and citation information see http://dx.doi.org/10.2196/jmir.37234

Table 3. BCT mappings detailed analysis per intervention

BCTs and BCCs	Grossman et al [61]	Hartman et al [62]	Park and Kim [60]	Cadmus-Bertram et al [64]	Finkelstein et al [65]	Fukuoka et al [66]	Lynch et al [67] Nguyen et al [68]	Anderson et al [73] McGuire et al [69]	Ryan et al [63]	Steinberg et al [48]	Im et al [70]	Im et al [71]	Ryan et al [72]	BCTs per category, n	Studies scoring ≥1 BCT, %
**1.Goals and planning,** n	4	5	1	3	0	4	4	3	5	2	0	0	3	34	77
BCTs from all possible BCTs (9 BCTs), %	44	5 6	11	33	0	44	44	33	56	22	0	0	33	117	29
1.1. Goal setting (behaviour)	X	X		X		X	X	X	X				X	8	63
1.2. Problem solving						X	X	X	X	X			X	6	46
1.3. Goal setting (outcome)	X	X												2	15
1.4. Action planning		X	X	X		X	X	X	X	X			X	9	69
1.5. Review behaviour goal(s)	X	X				X	X		X					5	38
1.6. Discrepancy between current behaviour and goal									X					1	8
1.7. Review	X	X		X										3	23

outcome goal(s)															
1.8. Behavioural contract														0	0
1.9. Commitment														0	0
**2.Feedback and monitoring,** n	4	2	4	3	2	2	2	1	2	4	0	0	2	28	85
BCTs from all possible BCTs (7 BCTs), %	57	29	57	43	29	29	29	14	29	57	0	0	29	91	31
2.1. Monitoring of behaviour by others without feedback														0	0
2.2. Feedback on behaviour	X	X	X	X	X	X	X	X	X	X			X	11	85
2.3. Self-monitoring of behaviour	X	X	X	X			X		X	X			X	8	62
2.4. Self-monitoring of outcome(s) of behaviour														0	0
2.5. Monitoring of outcome(s) of behaviour without feedback														0	0
2.6. Biofeedback	X		X							X				3	23
2.7. Feedback	X		X	X	X	X				X				6	46

on outcome(s) of behaviour															
**3. Social support,** n	1	0	0	0	0	1	1	1	1	2	2	1	1	11	69
BCTs from all possible BCTs (3 BCTs), %	33	0	0	0	0	33	33	33	33	67	67	33	33	39	28
3.1. Social support (unspecified)	X					X		X	X					4	31
3.2. Social support (practical)										X	X	X	X	4	31
3.3. Social support (emotional)							X			X	X			3	23
**4. Shaping knowledge**	1	1	1	1	1	1	0	1	1	1	1	1	1	12	92
BCTs from all possible BCTs (4 BCTs), %	25	25	25	25	25	25	0	25	25	25	25	25	25	52	23
4.1. Instruction on behaviour	X	X	X	X	X	X		X	X	X	X	X	X	12	92
4.2. Information about Antecedents														0	0
4.3. Re-attribution														0	0
4.4. Behavioural experiments														0	0
**5.Natural consequences**, n	0	0	0	0	0	1	1	1	0	0	1	1	0	5	38

BCTs from all possible BCTs (6 BCTs), %	0	0	0	0	0	17	17	17	0	0	17	17	0	78	6
5.1. Information about health consequences						X	X	X			X	X		5	38
5.2. Salience of consequences														0	0
5.3. Information about social and environmental consequences														0	0
5.4. Monitoring of emotional consequences														0	0
5.5. Anticipated regret														0	0
5.6. Information about emotional consequences														0	0
**6.Comparison of behaviour,** n	1	0	0	0	0	0	0	1	1	1	0	0	1	5	38
BCTs from all possible BCTs (3 BCTs), %	33	0	0	0	0	0	0	33	33	33	0	0	33	39	13
6.1. Demonstratio	X							X	X	X			X	5	38

n of the behaviour														
6.2. Social comparison													0	0
6.3. Information about others' approval													0	0
**7.Associations,** n	0	2	1	0	1	1	2	1	1	2	0	1	0	12	69
BCTs from all possible BCTs (8 BCTs), %	0	25	13	0	13	13	25	13	13	25	0	13	0	104	12
7.1. Prompts/cues		X	X		X	X	X	X	X	X		X		9	69
7.2. Cue signalling reward														0	0
7.3. Reduce prompts/cues		X					X			X				3	23
7.4. Remove access to the reward														0	0
7.5. Remove aversive stimulus														0	0
7.6. Satiation														0	0
7.7. Exposure														0	0
7.8. Associative learning														0	0
**8.Repetition and substitution,** n	3	1	5	1	2	3	2	3	3	0	2	2	3	30	92
BCTs from all	43	1	71	14	29	43	29	43	43	0	29	29	43	91	33

possible BCTs (8 BCTs), %		4													
8.1. Behavioural practice/rehearsal	X		X		X	X	X	X	X		X	X	X	10	77
8.2. Behaviour substitution			X											1	8
8.3. Habit formation	X		X		X	X	X	X	X		X	X	X	10	77
8.4. Habit reversal			X											1	8
8.5. Overcorrection			X											1	8
8.6. Generalisation of target behaviour														0	0
8.7. Graded tasks	X	X		X		X		X	X				X	7	54
**9.Comparison of outcomes,** n	1	1	1	0	0	0	0	1	1	0	0	1	0	6	46
BCTs from all possible BCTs (3 BCTs), %	33	3 3	33	0	0	0	0	33	33	0	0	33	0	39	15
9.1. Credible source	X	X	X					X	X			X		6	46
9.2. Pros and cons														0	0
9.3. Comparative imagining of future														0	0

outcomes															
**10.Reward and threat,** n	2	0	0	2	0	2	0	0	2	2	0	0	3	13	46
BCTs from all possible BCTs (11 BCTs), %	18	0	0	18	0	18	0	0	18	18	0	0	27	143	9
10.1. Material incentive (behaviour)	X			X		X			X	X			X	6	46
10.2. Material reward (behaviour)	X			X		X			X	X			X	6	46
10.3. Non-specific reward														0	0
10.4. Social reward														0	0
10.5. Social incentive														0	0
10.6. Non-specific incentive														0	0
10.7. Self-incentive														0	0
10.8. Incentive (outcome)														0	0
10.9. Self-reward														0	0
10.10. Reward (outcome)														0	0
10.11. Future punishment													X	1	8
**11.Regulation,** n	0	0	0	0	0	0	0	0	0	0	0	0	0	0	0

BCTs from all possible BCTs (4 BCTs), %	0	0	0	0	0	0	0	0	0	0	0	0	0	52	0
11.1. Pharmacological support														0	0
11.2. Reduce negative emotions														0	0
11.3. Conserving mental resources														0	0
11.4. Paradoxical instructions														0	0
**12.Antecedents,** n	3	1	1	1	1	1	1	0	0	0	0	0	1	10	62
BCTs from all possible BCTs (6 BCTs), %	50	1<br>7	17	17	17	17	17	0	0	0	0	0	17	78	13
12.1. Restructuring the physical environment	X													1	8
12.2. Restructuring the social environment														0	0
12.3. Avoidance/reducing exposure to cues for the behaviour														0	0
12.4.														0	0

Distraction															
12.5. Adding objects to the environment	X			X	X	X	X						X	6	46
12.6. Body changes	X	X	X											3	23
**13. Identity,** n	0	0	0	0	0	0	0	0	0	0	0	0	0	0	0
BCTs from all possible BCTs (5 BCTs), %	0	0	0	0	0	0	0	0	0	0	0	0	0	65	0
13.1. Identification of self as role model														0	0
13.2. Framing/reframing														0	0
13.3. Incompatible beliefs														0	0
13.4. Valued self-identify														0	0
13.5. Identity associated with changed behaviour														0	0
**14. Scheduled consequences**, n	1	0	0	1	0	0	0	0	0	0	0	0	1	3	23
BCTs from all possible BCTs (10 BCTs), %	10	0	0	10	0	0	0	0	0	0	0	0	10	130	2
14.1. Behaviour														0	0

cost														
14.2. Punishment													0	0
14.3. Remove reward												X	1	8
14.4. Reward approximation													0	0
14.5. Rewarding completion	X			X									2	15
14.6. Situation-specific reward													0	0
14.7. Reward incompatible behaviour													0	0
14.8. Reward alternative behaviour													0	0
14.9. Reduce reward frequency													0	0
14.10. Remove punishment													0	0
**15.Self-belief,** n	0	0	0	0	0	0	0	0	0	0	0	0	0	0	0
BCTs from all possible BCTs (4 BCTs), %	0	0	0	0	0	0	0	0	0	0	0	0	0	52	0
15.1. Verbal persuasion capability														0	0
15.2. Mental rehearsal of														0	0

successful performance														
15.3. Focus on past success													0	0
15.4. Self-talk													0	0
**16. Covert learning,** n	0	0	0	0	0	0	0	0	0	0	0	0	0	0	0
BCTs from all possible BCTs (3 BCTs), %	0	0	0	0	0	0	0	0	0	0	0	0	0	39	0
16.1. Imaginary punishment														0	0
16.2. Imaginary reward														0	0
16.3. Vicarious consequences														0	0
BCTs per study, n (%)	21 (23)	13 (14)	14 (15)	12 (13)	7 (8)	16 (17)	13 (14)	13 (14)	17 (18)	14 (15)	6 (6)	7 (8)	16 (17)	169 (14)	100

Each study was coded for BCT present (x) or absent [blank].
