# Supplementary material for: Behavior Change Techniques in Digital Health Interventions for Midlife Women: Systematic Review
Source: JMIR Mhealth Uhealth. 2022 Nov 9;10(11):e37234. doi: 10.2196/37234 (PMC9685514; doi:10.2196/37234)
Supplement: Multimedia Appendix 3 [file mhealth_v10i11e37234_app3.pdf]

This is a Multimedia Appendix to a full manuscript published in the JMIR mHealth and uHealth. For full copyright and citation information see http://dx.doi.org/10.2196/jmir.37234

**Table 5.** BCW mapping of all studies

| | | CAPABILITY | | | | | OPPORTUNITY | | MOTIVATION | | | | | | | |
|---|---|---|---|---|---|---|---|---|---|---|---|---|---|---|---|---|
| | | Physical | Psychological | | | | Social | Physical | Reflective | | | | | | Auto | |
| BCT Categories | Intervention Functions | Skills[a] | Know[b] | CogIS[c] | MAD[d] | BReg[e] | Soc infl[f] | Env res[g] | BCap[h] | BConi[i] | S/P ID[j] | Optim[k] | Intent[l] | Goals[m] | Reinf[n] | Em[o] |
| 1.Goals and planning | Enablement | | x | x | | x | | | x | x | x | | | x | | |
| 2.Feedback and monitoring | Enablement, Persuasion | | x | | | x | | | x | x | | x | | | x | x |
| 3. Social support | Enablement | | | | | | x | | | x | x | | x | | | x |
| 4. Shaping knowledge | Education | x | x | x | | | | | | | | | | | | |
| 5.Natural consequences | Enablement, Persuasion | | x | x | | | | | | x | | | | | | |
| 6.Comparison of behaviour | Training | x | x | | | | | x | | | | | | | | |
| 7.Associations | Env restr[p], Enablement | | | x | x | | x | x | | | | | | | | |
| 8.Repetition and substitution | Training, Skills | x | x | x | | x | | | | x | | | | | x | |
| 9.Compariso | Persuasion | | | | | | | | | x | | | | | | |

| | | | | | | | | | | | | | | | | |
|---|---|---|---|---|---|---|---|---|---|---|---|---|---|---|---|---|
| n of outcomes | | | | | | | | | | | | | | | | |
| 10.Reward and threat | Incentivisation, Coercion | | | | | | | | x | x | x | x | x | | x | |
| 11.Regulation | N/A | | | | | | | | | | | | | | | |
| 12.Antecedents | Env restr [p], Enablement | x | | | x | | | x | | | | | | | | |
| 13. Identity | N/A | | | | | | | | | | | | | | | |
| 14. Scheduled consequences | Incentivisation, Coercion | | | | | | | | x | | x | x | x | | x | X |
| 15.Self-belief | N/A | | | | | | | | | | | | | | | |
| 16. Covert learning | N/A | | | | | | | | | | | | | | | |
| TDF domains, n | | 8 | 13 | 10 | 3 | 15 | 3 | 5 | 6 | 10 | 7 | 4 | 4 | 3 | 4 | 3 |
| COM-B sub-components, n (%) | | 8 (8) | 41 (42) | | | | 3 (3) | 5 (5) | 34 (35) | | | | | | 7 (7) | |
| COM-B components, n (%) | | 49 (50) | | | | | 8 (8) | | 41 (42) | | | | | | | |

Each study was coded for BCT present (x) or absent [blank].

[a] Physical skills (Skills); [b] Knowledge (Know); [c] Cognitive and Interpersonal Skills (CogIS); [d] Memory, attention and decision processes (MAD); [e] Behavioural regulation (BReg); [f] Social influences (Soc infl); [g] Environmental context and resources (Env res); [h] Beliefs about capabilities (B Cap); [i] Beliefs about consequences (B Con); [j] Professional/Social role and identify (S/P ID); [k] Optimism (Optim); [l] Intentions (Intent); [m] Goals (Goals); [n] Reinforcement (Reinf); [o] Emotion (Em); [p] Environmental Restructuring (Env Restr)
