# Supplementary material for: Behavior Change Techniques in Digital Health Interventions for Midlife Women: Systematic Review
Source: JMIR Mhealth Uhealth. 2022 Nov 9;10(11):e37234. doi: 10.2196/37234 (PMC9685514; doi:10.2196/37234)
Supplement: Multimedia Appendix 4 [file mhealth_v10i11e37234_app4.pdf]

This is a Multimedia Appendix to a full manuscript published in the JMIR mHealth and uHealth. For full copyright and citation information see http://dx.doi.org/10.2196/jmir.37234

Table 6. TCS categories results for all studies

Theory	TCS category	TCS Items included	Max score per category, n	Description	Studies, n (%)	Mean score, n (SD)
Mentioned	Reference to underpinning theory (C1)	1, 2, 3	3	Stated or suggested rather than demonstrated theoretical base	10 (77)	1.69 (1.25)
Application	Targeting of relevant theoretical constructs (C2)	2, 5, 7, 8, 9, 10, 11	7	Targeted theoretical construct predicted behaviour; theory or predictors explicitly used for designing the intervention; the extent to which the intervention targets particular theory-relevant constructs	13 (100)	3.00 (1.53)
	Using theory to select recipients or tailor interventions (C3)	4, 6	2	Theory used to select participants; or tailor the intervention to the needs of a particular individual	3 (23)	0.23 (0.44)
	Measurement of constructs (C4)	12a, 12b	2	Measured theory-based constructs or predictors	9 (69)	1.23 (0.93)
Testing	Testing of theory: mediation effect (C5)	15, 16a, 16b, 16c, 16d, 17, 18	7	Measured theoretical constructs; the intervention changed the theoretical constructs; changes explain the effect	12 (92)	2.23 (1.36)
Refining	Refining theory (C6)	19a, 19b	2	Intervention results refined theory	3 (23)	0.23 (0.44)
	Totals		23			7.85 (3.87)

Composite scores were calculated for the six categories of theory used. An overall theory score for each included study was calculated as a sum of the total score with a maximum possible score of 23, representing 17 primary TCS items and six subitems.

Table 7. TCS item results for each study

TCS Item	Item category	Grossman et al [61]	Hartman et al [62]	Park and Kim [60]	Cadmus-Bertram et al [64]	Finkelstein et al [65]	Fukuoka et al [66]	Lynch et al [67] Nguyen et al [68]	Anderson et al [73] McGuire et al [69]	Ryan et al [63]	Steinberg et al [48]	Im et al [70]	Im et al [71]	Ryan et al [72]	TCS Items for all studies, n (%)
Theory mentioned (I1)	1	0	1	0	1	0	1	1	1	1	0	1	1	1	9 (69)
Targeted construct mentioned as predictor of behaviour (I2)	1 & 2	1	1	0	0	0	0	0	0	1	0	1	1	1	6 (46)
Intervention based on single theory (I3)	1	0	1	0	1	0	0	0	1	1	0	1	1	1	7 (54)
Theory or predictors used to select or develop recipients for the intervention (I4)	3	0	0	0	0	0	0	0	0	0	0	0	0	0	0 (0)
Theory or predictors used to select or develop intervention techniques (I5)	2	0	1	0	1	0	0	0	1	1	0	1	1	1	7 (54)
Theory or predictors used to tailor intervention techniques to recipients (I6)	3	0	0	1	0	0	1	0	0	1	0	0	0	0	3 (23)

All intervention techniques are explicitly linked to at least one theory relevant construct or predictor (I7)	2	0	0	0	0	0	0	0	0	0	0	0	0	0	0 (0)
At least one, but not all, of the intervention techniques are explicitly linked to at least one theory-relevant construct or predictor (I8)	2	1	1	0	0	0	0	0	0	1	0	1	1	1	6 (46)
Group of techniques are linked to a group of constructs or predictors (I9)	2	0	0	0	0	0	0	0	0	0	0	0	0	0	0 (0)
All theory-relevant constructs or predictors are explicitly linked to at least one intervention technique (I10)	2	0	0	0	0	0	0	0	0	1	0	1	1	1	4 (31)
At least one, but not all, of the theory relevant constructs are explicitly linked to at least one intervention technique (I11)	2	1	1	1	1	1	1	1	0	1	1	1	1	1	12 (92)
Theory-relevant constructs are	4	0	0	1	0	1	1	1	0	1	0	1	1	1	8 (62)

measured: post-intervention (I12a)															
Theory-relevant constructs are measured: post and pre intervention (I12b)	4	0	0	1	0	1	1	0	0	1	1	1	1	1	8 (62)
Changes in measured theory-relevant constructs (I15)	5	0	0	0	0	1	0	0	0	1	0	0	1	0	3 (23)
Mediator predicts the dependent variable (I16a)	5	0	0	0	0	1	0	1	1	1	1	1	1	1	8 (62)
Mediator predicts dependent variable, controlling for the independent variable (I16b)	5	0	0	0	0	0	0	0	0	0	0	0	0	0	0(0)
Intervention does not predict the dependent variable when controlling the independent variable (I16c)	5	0	0	0	0	0	0	0	0	0	0	0	0	0	0 (0)
Mediated effect is statistically significant (I16d)	5	0	0	1	1	1	1	1	1	1	0	0	1	0	8 (62)
Results discussed in relation to theory (I17)	5	1	0	1	0	0	1	1	0	1	0	0	1	1	7 (54)
Appropriate support for theory (I18)	5	0	0	0	1	0	1	0	0	1	0	0	0	0	3 (23)

Results used to refine theory: adding or removing constructs to the theory (I19a)	6	0	0	0	0	0	0	0	0	0	0	1	1	1	3 (23)
Results used to refine theory: specifying that the interrelationships between the theoretical constructs should be changed (I19b)	6	0	0	0	0	0	0	0	0	0	0	0	0	0	0 (0)
TCS items per study, n (%)		4 (17)	6 (26)	6 (26)	6 (26)	6 (26)	8 (35)	6 (26)	5 (22)	15 (65)	3 (13)	11 (48)	14 (61)	12 (52)	102 (34)

Each study was coded for TCS item present (1) or absent (0).
