# Supplementary material for: Behavior Change Techniques in Digital Health Interventions for Midlife Women: Systematic Review
Source: JMIR Mhealth Uhealth. 2022 Nov 9;10(11):e37234. doi: 10.2196/37234 (PMC9685514; doi:10.2196/37234)
Supplement: Multimedia Appendix 5 [file mhealth_v10i11e37234_app5.pdf]

This is a Multimedia Appendix to a full manuscript published in the JMIR mHealth and uHealth. For full copyright and citation information see http://dx.doi.org/10.2196/jmir.37234

Table 8. Summary of technological and non-technological components used in each study

		Technology used									
Intervention Type [a]	Study	Wearable	Web	App	Email	Peripheral Device	Phone/Text	Electronic doc	Face-to-face	Hard copy	Tech per study, n
Weight loss	Grossman et al [61]	x	x		x	x		x	x		6
Weight loss	Hartman et al [62]	x	x	x			x			x	5
Weight loss	Park and Kim [60]		x				x				2
Lifestyle (PA)	Cadmus-Bertram et al [64]	x	x							x	3
Lifestyle (PA)	Finkelstein et al [65]	x		x			x				3
Lifestyle (PA)	Fukuoka et al [66]	x		x			x		x	x	5
Lifestyle (PA, sleep)	Lynch et al [67] Nguyen et al [68]	x					x		x		3
Lifestyle (PA), Meno Sympt	McGuire et al [69] Anderson et al [73]		x					x	x	x	4
Lifestyle (Diet)	Ryan et al [63]		x	x			x		x	x	5
Lifestyle (Diet)	Steinberg et al [48]		x	x			x			x	4
Meno sympt	Im et al [70]		x		x						2

Meno sympt	Im et al [71]		x		x						2
Meno sympt	Ryan et al [72]	x		x			x				3
Tech component across studies, n (%)		7(54)	9(69)	6(46)	3(23)	1(8)	8(62)	2(15)	5(38)	6(46)	

Each study was coded for technology present (x) or absent [blank].

[a] Intervention types included 1) Weight loss 2) Lifestyle physical activity (PA), 3) Lifestyle (Diet), 4) Lifestyle (Sleep), 5) Menopausal symptoms (Meno sympt).
