# Supplementary material for: Behavior Change Techniques in Digital Health Interventions for Midlife Women: Systematic Review
Source: JMIR Mhealth Uhealth. 2022 Nov 9;10(11):e37234. doi: 10.2196/37234 (PMC9685514; doi:10.2196/37234)
Supplement: Multimedia Appendix 6 [file mhealth_v10i11e37234_app6.pdf]

This is a Multimedia Appendix to a full manuscript published in the JMIR mHealth and uHealth. For full copyright and citation information see http://dx.doi.org/10.2196/jmir.37234

Table 9. Treatment fidelity results for all studies

Fidelity domains	Grossman et al [61]	Hartman et al [62]	Park and Kim [60]	Cadmus-Bertram et al [64]	Finkelstein et al [65]	Fukuoka et al [66]	Lynch et al [67] Nguyen et al [68]	Anderson et al [73] McGuire et al [69]	Ryan et al [63]	Steinberg et al [48]	Im et al [70]	Im et al [71]	Ryan et al [72]	Mean proportion (SD)	Median proportion
1. Treatment design	0.63	0.38	0.19	0.31	0.19	0.69	0.44	0.38	0.75	0.38	0.50	0.50	0.56	0.45 (0.18)	0.44
2. Training providers	0.14	0.57	0.14	0.14	0.00	0.14	0.43	0.57	0.71	0.43	0.29	0.29	0.57	0.34 (0.22)	0.29
3. Delivery	0.22	0.33	0.11	0.33	0.11	0.33	0.56	0.33	0.33	0.44	0.22	0.22	0.56	0.32 (0.14)	0.33
4. Receipt	0.00	0.50	0.00	0.50	0.00	0.00	0.00	0.25	0.50	0.25	0.40	0.20	0.75	0.26 (0.25)	0.25
5. Enactment	0.50	0.50	0.50	0.50	0.50	0.50	0.50	0.50	0.50	0.50	0.50	0.50	0.50	0.50 (0.00)	0.50
Overall fidelity proportion per study	0.38	0.43	0.16	0.32	0.14	0.43	0.43	0.41	0.62	0.41	0.39	0.37	0.59	0.39 (0.14)	0.41

Table 10. Treatment fidelity for each study

Fidelity categories	Grossman et al [61]	Hartman et al [62]	Park and Kim [60]	Cadmus-Bertram et al [64]	Finkelstein et al [65]	Fukuoka et al [66]	Lynch et al [67] Nguyen et al [68]	Anderson et al [73] McGuire et al [69]	Ryan et al [63]	Steinberg et al [48]	Im et al [70]	Im et al [71]	Ryan et al [72]	Totals per domain, n (%)
**Treatment design, n (%)**	10 (63)	6 (38)	3 (19)	5 (31)	3 (19)	11 (69)	7 (44)	6 (38)	12 (75)	6 (38)	8 (50)	8 (50)	9 (56)	94 (45)
1. Intervention dose information (Intervention Group)														
a. Length of contact (minutes)	1	0	0	0	0	0	0	0	1	0	0	0	0	
b. Number of contacts	1	1	0	1	0	1	1	1	1	1	1	1	1	
c. Intervention content	1	1	1	1	1	1	1	1	1	1	1	1	1	
d. Duration of contact over time	1	0	0	0	0	0	0	0	1	0	0	0	0	

2. Intervention dose information (Comparison / Control Group)														
a. Length of contact (minutes)	1	0	0	0	0	0	0	0	1	0	0	0	0	
b. Number of contacts	1	1	0	1	0	0	1	1	1	1	1	1	1	
c. Intervention content	1	1	1	1	1	1	1	1	1	1	1	1	1	
d. Duration of contact over time	1	0	0	0	0	0	0	0	1	0	0	0	0	
e. Method to ensure that dose is equivalent between conditions.	0	0	0	0	0	0	0	0	0	0	0	0	0	
f. Method to ensure that dose is equivalent for participants within conditions	0	0	0	0	0	0	0	0	0	0	0	0	0	
3. Specification of provider credentials that are needed.	1	1	0	0	0	1	1	1	1	1	1	1	1	
4. Theoretical model upon which the intervention is based is clearly articulated.														
a. The active ingredients are specified and incorporated into the intervention	1	1	1	1	1	1	1	1	1	1	1	1	1	
b. Use of experts or protocol review group to determine whether the intervention protocol reflects the underlying theoretical model or clinical guidelines	0	0	0	0	0	1	1	0	1	0	1	1	1	
c. Plan to ensure that the measures	0	0	0	0	0	0	0	0	1	0	1	1	1	

reflect the hypothesized theoretical constructs/mechanisms of action														
5. Potential confounders that limit the ability to make conclusions at the end of the trial are identified?	0	0	0	0	0	0	0	0	0	0	0	0	0	
6. Plan to address possible setbacks in implementation (i.e., back-up systems or providers)	0	0	0	0	0	0	0	0	0	0	0	0	1	
6. If more than one intervention is described, all described equally well.	NA	NA	NA	NA	NA	NA	NA	NA	NA	NA	NA	NA	NA	
**Training Providers, n (%)**	1 (14)	4 (57)	1 (14)	1 (14)	0 (0)	1 (14)	3 (43)	4 (57)	5 (71)	3 (43)	2 (29)	2 (29)	4 (57)	31 (34)
1. Description of how providers will be trained (manual of training procedures)	0	0	0	1	0	0	1	1	1	1	1	1	1	
2. Standardization of provider training (especially if multiple waves of training are needed for multiple groups of providers)	0	1	0	0	0	0	1	1	1	1	1	1	0	
3. Assessment of provider skill acquisition.	0	1	0	0	0	0	0	0	1	0	0	0	1	

4. Assessment and monitoring of provider skill maintenance over time	0	1	0	0	0	0	0	1	1	0	0	0	1	
5. Characteristics being sought in a treatment provider are articulated a priori. Characteristics that should be avoided in a treatment provider are articulated a priori.	0	0	0	0	0	0	0	0	0	0	0	0	0	
6. At the hiring stage, assessment of whether or not there is a good fit between the provider and the intervention (e.g., ensure that providers find the intervention acceptable, credible and potentially efficacious	1	1	1	0	0	1	1	1	1	1	0	0	1	
7. There is a training plan that takes into account trainees' different education and experience and learning styles	0	0	0	0	0	0	0	0	0	0	0	0	0	
**Delivery of Treatment, n (%)**	2 (22)	3 (33)	1 (11)	3 (33)	1 (11)	3 (33)	5 (56)	3 (33)	3 (33)	4 (44)	2 (22)	2 (22)	5 (56)	37 (32)
1. Method to ensure that the content of the	0	0	0	0	0	0	1	0	1	1	0	0	1	

intervention is delivered as specified.														
2. Method to ensure that the dose of the intervention is delivered as specified.	0	0	0	0	0	0	1	0	0	1	0	0	0	
3. Mechanism to assess if the provider actually adhered to the intervention plan or in the case of computer delivered interventions, method to assess participants' contact with the information.	1	1	0	1	0	1	1	1	0	1	0	0	1	
4. Assessment of non-specific treatment effects.	1	1	1	1	1	1	1	1	1	1	1	1	1	
5. Use of treatment manual.	0	1	0	1	0	0	0	1	1	0	1	1	1	
6. There is a plan for the assessment of whether or not the active ingredients were delivered.	0	0	0	0	0	0	1	0	0	0	0	0	1	
7. There is a plan for the assessment of whether or not proscribed components were delivered. (e.g., components that are unnecessary or unhelpful)	0	0	0	0	0	0	0	0	0	0	0	0	0	

8. There is a plan for how will contamination between conditions be prevented.	0	0	0	0	0	0	0	0	0	0	0	0	0	
9. There is an a priori specification of treatment fidelity (e.g., providers adhere to delivering >80% of components).	0	0	0	0	0	1	0	0	0	0	0	0	0	
**Receipt of Treatment, n**	0 (0)	2 (50)	0 (0)	2 (50)	0 (0)	0 (0)	0 (0)	1 (25)	2 (50)	1 (25)	2 (40)	1 (20)	3 (75)	14 (26)
1. There is an assessment of the degree to which participants understood the intervention.	0	0	0	0	0	0	0	0	0	1	0	0	0	
2. There are specification of strategies that will be used to improve participant comprehension of the intervention.	0	0	0	1	0	0	0	1	1	0	0	0	1	
3. The participants' ability to perform the intervention skills will be assessed during the intervention period.	0	1	0	1	0	0	0	0	1	0	0	0	1	
4. A strategy will be used to improve subject performance of intervention skills during the intervention period.	0	1	0	0	0	0	0	0	0	0	1	0	1	

5. Multicultural factors considered in the development and delivery of the intervention (e.g., provided in native language; protocol is consistent with the values of the target group)	NA	NA	NA	NA	NA	NA	NA	NA	NA	NA	1	1	NA	
**Enactment of Treatment Skills, n**	1 (50)	1 (50)	1 (50)	1 (50)	1 (50)	1 (50)	1 (50)	1 (50)	1 (50)	1 (50)	1 (50)	1 (50)	1 (50)	13 (50)
1. Participant performance of the intervention skills will be assessed in settings in which the intervention might be applied.	1	1	1	1	1	1	1	1	1	1	1	1	1	
2. A strategy will be used to assess performance of the intervention skills in settings in which the intervention might be applied.	0	0	0	0	0	0	0	0	0	0	0	0	0	
Totals per study, n (%)	14 (38)	16 (43)	6 (16)	12 (32)	5 (14)	16 (43)	16 (43)	15 (41)	23 (62)	15 (41)	15 (39)	14 (37)	22 (59)	

Each study was coded for treatment fidelity item present (1) or absent (0).
